# Cost-effectiveness analysis of rezvilutamide versus bicalutamide and androgen-deprivation therapy in patients with high-volume, metastatic, hormone-sensitive prostate cancer: a Markov’s model-based evaluation

**DOI:** 10.3389/fpubh.2025.1574780

**Published:** 2025-05-27

**Authors:** Juan Hong, Xiaohui Zeng, Wenjun Chen, Ziyuan Zhou, Yuming He, Jun Meng, Lihui Ouyang

**Affiliations:** ^1^Department of Pharmacy, National Cancer Center/National Clinical Research Center for Cancer/Cancer Hospital & Shenzhen Hospital, Chinese Academy of Medical Sciences and Peking Union Medical College, Shenzhen, China; ^2^Department of Nuclear Medicine/PET Image Center, The Second Xiangya Hospital of Central South University, Changsha, China; ^3^School of Pharmacy, Guangdong Medical University, Dongguan, China; ^4^Department of Pharmacy, The Affiliated Changsha Hospital of Xiangya School of Medicine, Central South University, Changsha, China

**Keywords:** cost-effectiveness, rezvilutamide, bicalutamide, high-volume mHSPC, Markov model

## Abstract

**Objectives:**

Rezvilutamide, an androgen-receptor inhibitor, has been approved by the Chinese National Medical Products Administration as a first-line treatment for high-volume metastatic hormone-sensitive prostate cancer (mHSPC). This study aims to assess the cost-effectiveness of rezvilutamide plus androgen-deprivation therapy (ADT) compared to bicalutamide plus ADT for the first-line treatment of high-volume mHSPC in China.

**Methods:**

A Markov model with three health states was developed to evaluate the health and economic outcomes of first-line treatment for high-volume mHSPC. Efficacy data were sourced from the CHART trial. Quality-adjusted life-years (QALYs) and incremental cost-effectiveness ratios (ICERs) were calculated. To address modeling uncertainties, one-way sensitivity analysis and probabilistic sensitivity analysis were performed.

**Results:**

Compared with bicalutamide plus ADT, rezvilutamide plus ADT resulted in an additional 2.16 QALYs, with an ICER of $39,122.16/QALY. At a willingness-to-pay (WTP) threshold of three times the gross domestic product per capita in China for 2023 ($37,256.3/QALY), the probability of cost-effectiveness for rezvilutamide plus ADT was 30%. One-way sensitivity analysis revealed that the results were most sensitive to the cost of rezvilutamide. Scenario analysis indicated that rezvilutamide could be considered cost-effective if priced below $705.46 per cycle.

**Conclusion:**

From the perspective of Chinese payers, rezvilutamide plus ADT appears to be a less cost-effective strategy compared to bicalutamide plus ADT for the first-line treatment of high-volume mHSPC in China.

## Introduction

Prostate cancer is a prevalent genitourinary malignancy, particularly among older men. According to the Global Cancer Statistics 2022, prostate cancer is the second most frequently diagnosed malignancy in men worldwide and the fifth leading cause of cancer-related deaths among men (1). In China, it accounts for 13.42% of male cancer incidence and 4.75% of cancer-related deaths (2). Although the 5-year survival rate for clinically localized prostate cancer in China increased from 53.8% to 66.4% between 2003 and 2015, it remained significantly lower compared to developed countries such as the United States, where the survival rate was close to 100% (3). However, only 30% of cases are diagnosed at an early stage, while the majority of prostate cancer cases in China are diagnosed in the intermediate to advanced stages (3). The prognosis for metastatic prostate cancer is generally poor, with a 5-year survival rate of approximately 36.6% (4, 5). As a result, innovative therapeutic approaches are critically needed to improve survival outcomes for patients with metastatic prostate cancer.

For many years, androgen deprivation therapy (ADT), which includes both surgical and chemical castration, has been the standard of care for advanced prostate cancer (6). However, most patients with metastatic hormone-sensitive prostate cancer (mHSPC) who received ADT alone are at risk of developing metastatic castration-resistant prostate cancer (mCRPC) within 2 years (7). Recent advances have revealed the considerable potential of combining docetaxel with ADT. This combination therapy has demonstrated remarkable superiority over ADT alone (8–10). Bicalutamide, a first-generation androgen receptor (AR) inhibitor approved in the United States in 1995, has been commonly used for metastatic prostate cancer. However, first-generation AR inhibitors exhibit weak affinity for the AR, leading to limited efficacy in blocking AR activity and potential drug resistance due to AR overexpression or mutation (11). Consequently, first-generation AR inhibitors in combination with ADT, as well as ADT alone, were not recommended as standard treatment options for mHSPC in the 2021 European Association of Urology Guidelines (12). Second-generation AR inhibitors, such as enzalutamide (approved in the US in 2012), apalutamide (approved in 2018), and darolutamide (approved in 2019), have been shown to effectively delay the onset of castration resistance and improve overall survival for patients (13–16). In June 2022, the Chinese National Medical Products Administration (NMPA) approved rezvilutamide for the treatment of high-volume mHSPC. This treatment approach is also recommended by the Chinese Society of Clinical Oncology (CSCO) diagnosis and treatment of prostate cancer guideline (17).

The clinical efficacy of rezvilutamide in combination with ADT was evaluated in the CHART study (18). CHART was a randomized, open-label, phase 3 study conducted across 72 hospitals in China, Poland, the Czech Republic, and Bulgaria. The study aimed to assess the efficacy and safety of rezvilutamide plus ADT as a first-line therapy for patients with high-volume mHSPC who had not previously received chemotherapy or other localized treatments. Total of 654 patients were eligible and randomly assigned to either the rezvilutamide group (240 mg orally once daily in a 4-week cycle; *n* = 326) or the bicalutamide group (50 mg orally once daily in a 4-week cycle; *n* = 326). All patients received background therapy with either surgical ADT or luteinizing hormone-releasing hormone (LHRH) agonists or antagonists, in accordance with the package insert, throughout the study period. The co-primary endpoints of the study were radiographic progression-free survival (rPFS) and overall survival (OS). The results showed that the rezvilutamide group significantly improved rPFS compared with the bicalutamide group [not reached vs. 25.1 months; hazard ratio = 0.44, 95% confidence interval (CI) 0.33–0.58, *p* < 0.0001]. Furthermore, rezvilutamide significantly improved OS compared to bicalutamide (not reached vs. not reached; hazard ratio = 0.58, 95% CI 0.44–0.77, *p* < 0.0001).

The findings of the CHART trial formed the basis for the regulatory approval of rezvilutamide in combination with ADT for the treatment of high-volume mHSPC in China. However, clinical treatment decisions and national health policy require evidence of cost-effectiveness. Since existing economics assessment studies have used partitioned survival model and reached controversial conclusions (19, 20). The objective of this study conducted a Markov model to evaluate the cost-effectiveness of rezvilutamide plus ADT versus bicalutamide plus ADT for high-volume mHSPC, based on the data from the CHART trial, from the perspective of the Chinese healthcare system.

## Methods

### Model overview

**Figure 1:**
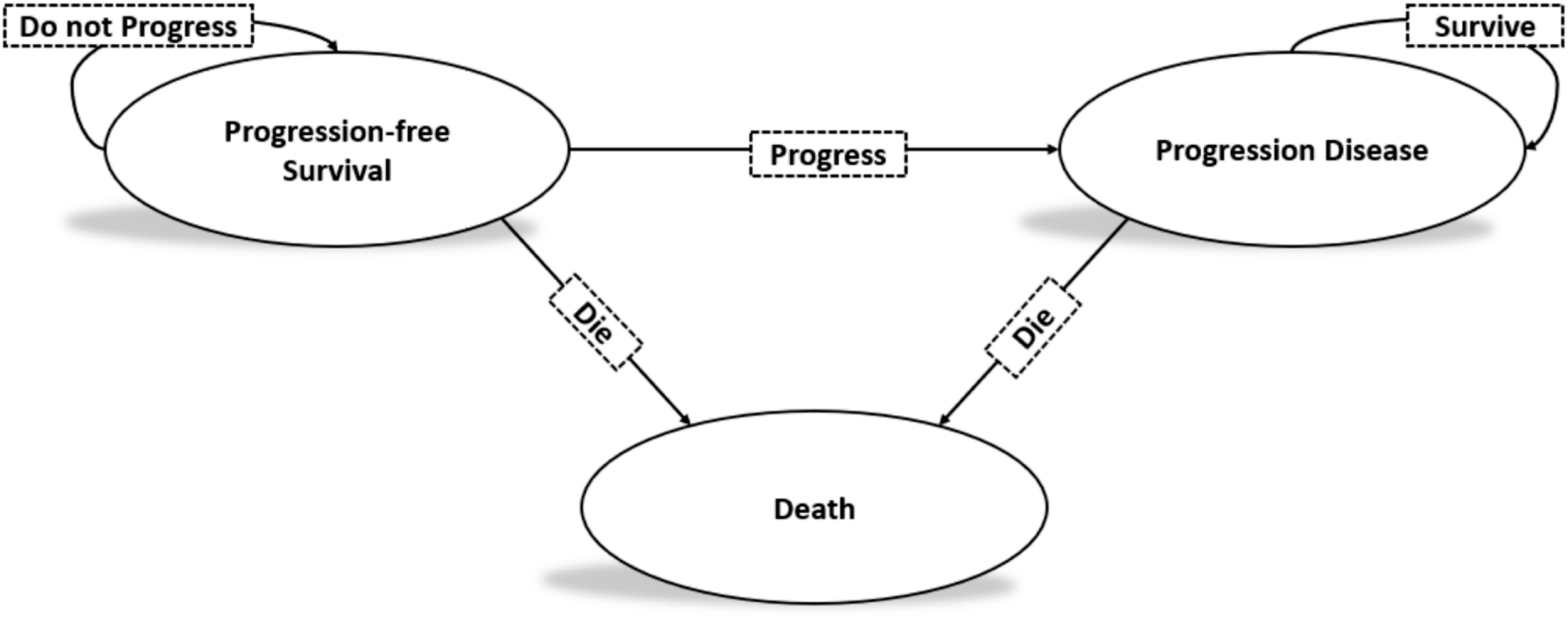
The Markov state transition model.

This study adhered to the Consolidated Health Economic Evaluation Reporting Standards (CHEERS) reporting guidelines (21, 22). A Markov model was developed to simulate the costs and health benefits associated with the treatment of high-volume mHSPC using rezvilutamide plus ADT versus bicalutamide plus ADT. The model included three distinct health states: progression-free survival (PFS), progressive disease (PD), and death (Figure 1). The cycle length of the Markov model was set to 28 days, aligning with the treatment periods. The average life expectancy in China in 2020 was 77.93 years (23). Given that the median age of patients in the CHART study was 69.2 years (range: 64–74 years), a 13-year time horizon was selected to better represent long-term survival of patients. All patients began in the PFS state and transitioned to either the PD state or death. Patients in the PD state could remain there or transition to death. The model’s outcomes included life years, quality-adjusted life years (QALYs), and costs. According to Chinese pharmacoeconomic evaluation guidelines, both costs and QALYs were discounted at an annual rate of 5%. Incremental cost-effectiveness ratios (ICERs) were calculated to represent the cost per additional QALY gained. The cost-effectiveness threshold in China was defined as $37,256.3, equivalent to three times the per capita gross domestic product (GDP) of China in 2023 (24, 25).

### Clinical efficacy

Survival parameters were primarily derived from the Kaplan–Meier (KM) curve of the CHART trial (18). We used the Engauge Digitizer software to extract digitized data points from the PFS and OS KM curves of the CHART study. Individual patient data were then reconstructed using standard statistical methods as outlined by Guyot et al. (26). Several parametric survival functions were evaluated, including the Exponential, Weibull, Log-logistic, Gompertz, and Log-normal distributions. The model selection was based on the Akaike information criterion (AIC), Bayesian information criterion (BIC), and visual inspection of the parametric extrapolation and long-term survival estimates. The distribution with the lowest AIC or BIC values was chosen as the best-fit model (27).

### Cost estimates

This analysis focused solely on direct medical costs, which included drug costs, costs for managing treatment-related adverse events (AEs), subsequent treatment costs, and disease management costs (outpatient visits, supportive care, nursing, laboratory tests). Drug costs were estimated based on patient dosing schedules and unit prices, calculated per treatment cycle. Unit drug costs were sourced from Hunan Public Resources Trading Service Platform (28). Costs associated with subsequent treatments, AE management, and end-of-life care were either estimated or referenced from published literature (29–31). For AE management costs, only grade ≥ 3 events from the CHART trials were included (18). According to the CHART study, the incidence of treatment-emergent AEs was 7% for hypertension and 2% for hypertriglyceridemia in the rezvilutamide plus ADT group, compared to 8% and 7%, respectively, in the bicalutamide plus ADT group. Upon disease progression, 27 and 62% of patients received subsequent treatments, including hormonal therapy, abiraterone acetate, enzalutamide, and docetaxel; the others received supportive care. All costs were adjusted to US dollars for 2024. Chinese Yuan was converted to US dollars using the exchange rate formula: 1 US $ = 7.1954 CNY (32). Key costs are shown in Table 1.

**Table 1:** Model parameters of clinical data, costs and utilities: baseline values, ranges, and distributions.

Unit	Baseline value (range)	Distribution	Source
Survival model for rezvilutamide			
PFS	AIC = −111.95, BIC = −109.12	Log-normal	Estimated
OS	AIC = −268.95, BIC = −263.40	Log-normal	Estimated
Survival model for bicalutamide			
PFS	AIC = −80.94, BIC = −77.96	Log-normal	Estimated
OS	AIC = −329.31, BIC = −323.51	Log-normal	Estimated
Rezvilutamide	823.03(658.42–987.63)	Gamma	Local database
Bicalutamide	120.75(96.60–144.90)	Gamma	Local database
Goserelin	149.23(119.38–179.07)	Gamma	Local database
Leuprolide	176.86(141.49–212.23)	Gamma	Local database
Triptorelin	163.90(131.12–196.68)	Gamma	Local database
Degarelix	104.23(83.39–125.08)	Gamma	Local database
Average	148.55(118.84–178.26)	Gamma	Estimated
Subsequent antitumor therapy			
Abiraterone	177.89(142.31–213.47)	Gamma	Local database
Enzalutamide	1083.36(866.69–1300.03)	Gamma	Local database
Docetaxel	815.74(652.59–978.89)	Gamma	Local database
Prednisone	0.49(0.39–0.59)	Gamma	Local database
Apalutamide	917.25(733.80–1100.70)	Gamma	Local database
Darolutamide	1044.00(835.20–1252.80)	Gamma	Local database
Surgical ADT	1389.78(1111.82–1667.73)	Gamma	Estimated
Cost of routine treatment and Checklist per unit	642.89(514.32–771.47)	Gamma	Estimated
Testosterone concentrations	4.17(3.34–5.00)	Gamma	Estimated
Prostate specific antigen	16.68(13.34–20.01)	Gamma	Estimated
CT	27.80(22.24–33.35)	Gamma	Estimated
Cost of supportive care per cycle	117.1(93.68–140.52)	Gamma	(28)
Routine follow-up of patients per unit	51.5(41.2–61.8)	Gamma	Estimated
AE			
Hypertension	12.15(9.72–14.58)	Gamma	(8)
Hypertriglyceridemia	13.23(10.58–15.88)	Gamma	(8)
Health state utility			
PFS	0.76(0.684–0.836)	Beta	(31)
PD	0.68(0.612–0.748)	Beta	(31)
Discount rate	0.05(0.00–0.08)	Beta	(22)

AIC, Akaike information criterion; BIC, Bayesian information criterion; OS, overall survival; PFS, progression-free survival. ADT, androgen deprivation therapy; AE, adverse event.

### Utility estimates

Quality-adjusted life-years were estimated by adjusting survival time with health-related quality of life. Separate health state utility (HSU) values were applied for patients in the PFS and PD states. The HSU values for these different health states in the CHART trial population were derived from published literature (33). The values and sources for HSU were detailed in Table 1.

### Sensitivity analysis

The robustness of the results was tested with a series of one-way sensitivity analysis on several parameters. In the one-way sensitivity analysis, the cost parameters ranged between −20% and +20% and the utility parameters were variable at 10% efficiency, as detailed in Table 1. The results of the one-way sensitivity analysis are presented as tornado diagrams. Moreover, probabilistic sensitivity analysis were conducted using Monte Carlo simulations with 10,000 replicated outcomes. The results of these are presented as cost-effectiveness acceptability curves. For the model, different distribution types were applied to each parameter: Gamma distributions for cost inputs and Beta distributions for utility values.

## Results

### Base-case analysis

The results of the deterministic analysis are showed in Table 2. In patients with high-volume mHSPC, compared to bicalutamide plus ADT, rezvilutamide plus ADT yielded an additional 2.16 QALYs (5.46 QALYs vs. 3.31 QALYs), corresponding to an incremental cost of $84,417.48. The calculated ICER was $39,122.16/QALY gained, which exceeded the WTP threshold of three times the GDP per capita in China.

**Table 2:** Summary of results of the base-case analysis.

Regimens	Costs ($)	Incremental Cost ($)	QALYs	Incremental QALYs*	ICER*
Rezvilutamide	126510.14	84417.48	5.46	2.15	39122.16
Bicalutamide	42092.66	NA	3.31	NA	NA

QALYs, Quality-adjusted life years; ICER, Incremental cost-effectiveness ratio.

### Sensitivity analysis

**Figure 2:**
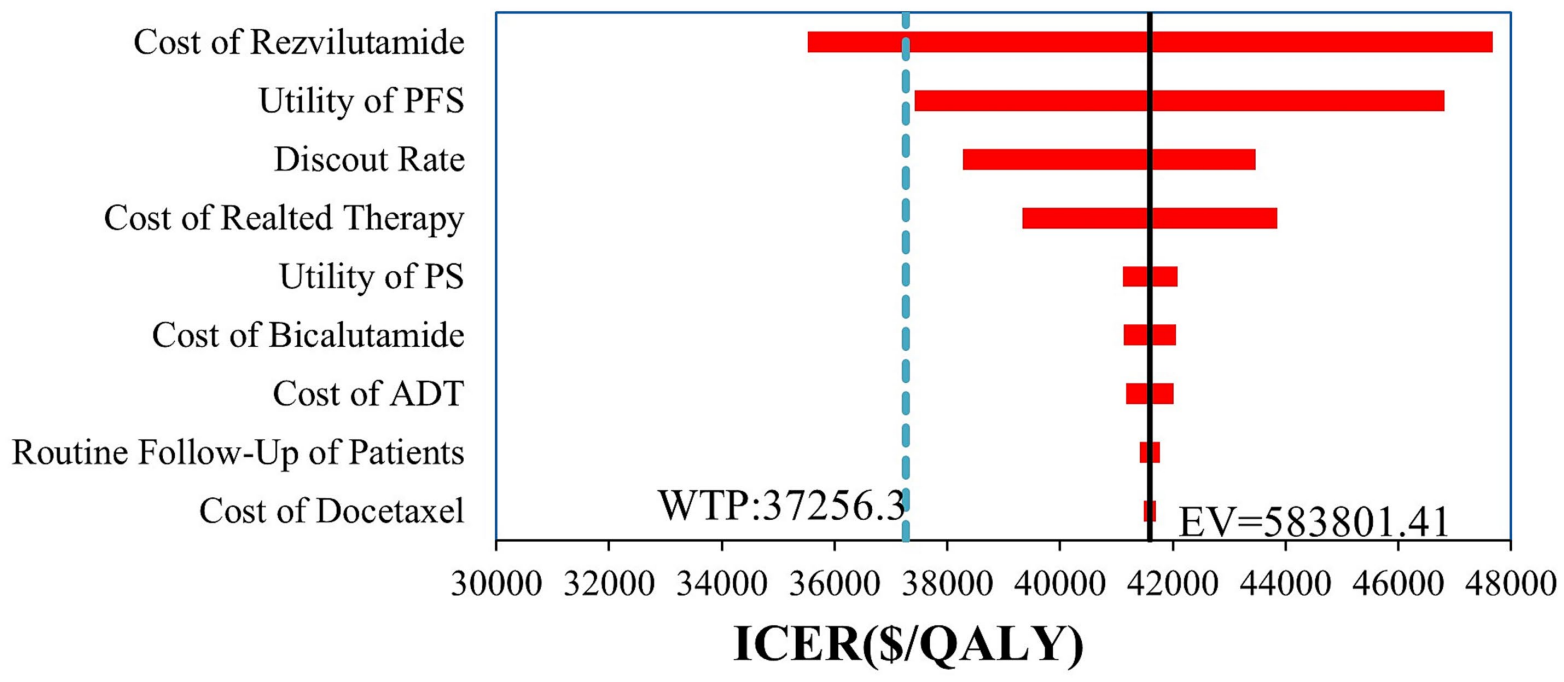
Tornado plot generated in the one-way deterministic sensitivity analysis. Only the top 9 most influential parameters are presented. PFS, progression-free survival; PS, progression survival; ADT, androgen deprivation therapy; WTP, willingness-to-pay; QALY, quality-adjusted life-year; ICER incremental cost-effectiveness ratio; EV, expect value.

**Figure 3:**
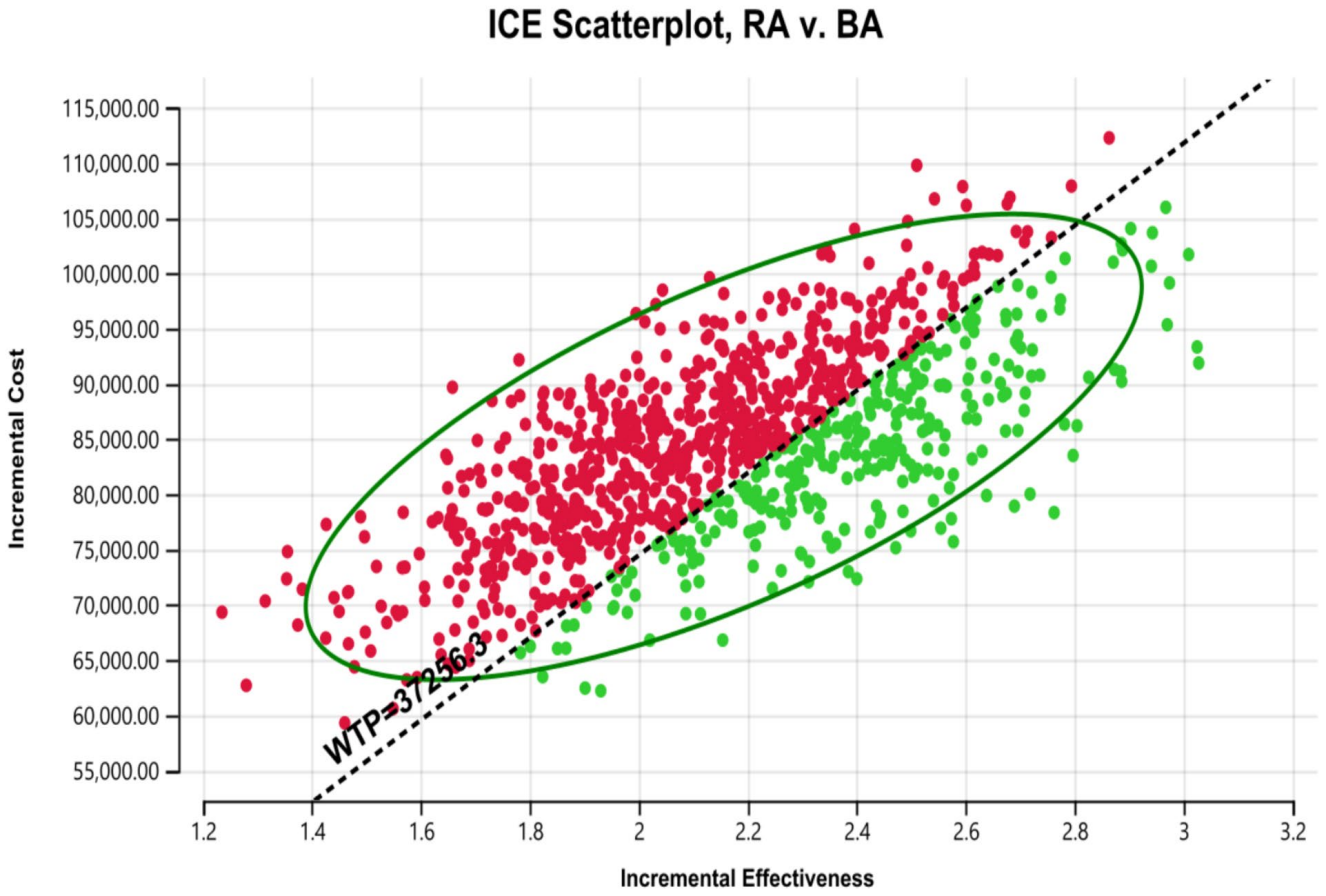
Cost-effectiveness plane generated in the probabilistic sensitivity analysis. WTP, willingness to pay; RA, rezvilutamide; BA, bicalutamide. Red: ICER value greater than WTP. Green: ICER value less than WTP.

**Figure 4:**
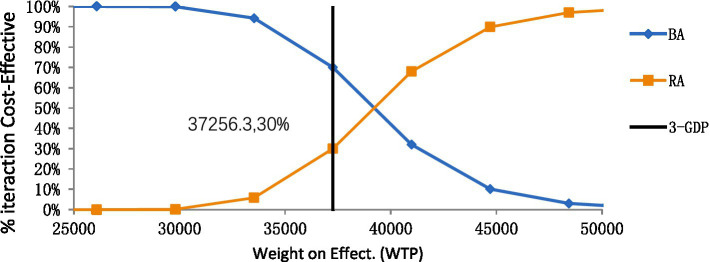
The cost-effectiveness acceptability curve for rezvilutamide versus bicalutamide. BA, bicalutamide; RA, rezvilutamide; WTP, willingness-to-pay; GDP, gross domestic product.

According to the one-way deterministic sensitivity analysis (DSA), the model was most sensitive to the cost of rezvilutamide (Figure 2). In the probabilistic sensitivity analysis (PSA), the cost-effectiveness plane illustrated the results of 10,000 Monte Carlo simulations (Figure 3). Comparing rezvilutamide plus ADT with bicalutamide plus ADT the cost-effectiveness acceptability curve indicated a nearly 30% probability of cost-effectiveness at the WTP threshold of $37,256.30, consistent with the base-case analysis results (Figure 4).

## Discussion

The introduction of second-generation AR inhibitors has brought a new ray of hope to the treatment of high-volume mHSPC. The combination of AR inhibitors with ADT has been recommended as a first-line treatment in the guideline of the CSCO for prostate cancer (17). The recommended first-line treatment for patients with high-volume mHSPC includes ADT combined with an antiandrogen, such as bicalutamide, rezvilutamide, abiraterone, enzalutamide, or apalutamide. Despite these positive developments, many of these second-generation AR inhibitors were imported medications, and the clinical studies that have been registered primarily focused on Western populations. The CHART trial demonstrated that rezvilutamide, in combination with ADT, significantly improved the prognosis of patients with high-volume mHSPC when compared with bicalutamide plus ADT (18).

The purpose of this study, which employed a Markov model, was to assess the cost-effectiveness of rezvilutamide plus ADT compared with bicalutamide plus ADT in the treatment of high-volume mHSPC. The results suggest that, compared with bicalutamide plus ADT, rezvilutamide plus ADT was associated with an incremental survival of 2.16 QALYs and an incremental cost of $84,417.28. The calculated ICER was $39,122.16 per QALY. The cost-effectiveness acceptability curve revealed that rezvilutamide plus ADT was not cost-effective when the WTP threshold was set at $37,256.3 per QALY. However, these findings should not be used as a basis for limiting the use of rezvilutamide, as this may result in missed opportunities for beneficial treatment options. Instead, they should be regarded as economic considerations for informing the implementation of China’s national pricing negotiation policies. To address the issue of high drug prices, promote patient access, and ensure the sustainability of the medical insurance fund, China formally launched national reimbursement-linked price negotiations in 2017 (34, 35). Such a policy could significantly improve the cost-effectiveness of rezvilutamide.

The results of the one-way sensitivity analysis showed that the cost of rezvilutamide was the most sensitive factor affecting the ICER. The analysis revealed that the cost-effectiveness of rezvilutamide plus ADT compared to bicalutamide plus ADT could be influenced by the price of rezvilutamide. Rezvilutamide can be considered cost-effective only when priced below $705.46 per cycle at a WTP threshold of $37,256.3 per QALY. The results of both the one-way sensitivity analysis and probabilistic sensitivity analysis demonstrate the robustness of these findings. These results provide important insights for China’s health insurance policymakers when determining the price of rezvilutamide following its launch.

Several studies have assessed the cost-effectiveness of first-line treatments for high-volume mHSPC, including two Chinese studies focusing on the cost-effectiveness of rezvilutamide. As a novel therapy, rezvilutamide was associated with a high economic burden, highlighting the need for pharmacoeconomic research based on the CHART trial to evaluate its cost-effectiveness (19, 20, 36, 37). Ding et al. (20) previously conducted a partitioned survival model to evaluate the cost-effectiveness of rezvilutamide combined with ADT for high-volume mHSPC in China. Their study demonstrated that rezvilutamide plus ADT was more cost-effective compared to bicalutamide plus ADT as the first-line treatment for high-volume mHSPC from the perspective of the Chinese healthcare system (20). In contrast, Wu et al. (19) conducted a similar study using a partitioned survival model and concluded that rezvilutamide plus ADT is unlikely to be cost-effective for most adults when compared to bicalutamide plus ADT, considering a WTP threshold of $38,223.3 per QALY from the perspective of the Chinese healthcare system. However, they suggested that a promising economic advantage could be achieved if rezvilutamide were included in the National Reimbursement Drug List (NRDL) with a 10% price reduction (19). Due to the discrepancies between the findings of these two studies, we constructed a Markov model to assess the cost-effectiveness of rezvilutamide plus ADT versus bicalutamide plus ADT as a first-line treatment for high-volume mHSPC from the perspective of the Chinese healthcare system. Our results indicated that rezvilutamide treatment regimen would be considered cost-effective only when priced below $705.46 per cycle at a WTP threshold of $37,256.3 per QALY, yielding findings consistent with those of Wu et al. (19). This provided new evidence to inform clinical decision-making regarding antiandrogen drugs for Chinese patients with high-volume mHSPC based a Markov model cost-effectiveness comparison.

The present study has several limitations. First, the pharmacoeconomic evaluation was based on the CHART trial, which unfortunately had a limited follow-up period. As a result, we obtained progression-free survival PFS and OS data by fitting parameter distributions. Although extrapolation could obtain relevant data outside the follow-up period of the CHART trial, this would increase model uncertainty. To mitigate this, we conducted a comparative analysis using the Akaike Information Criterion (AIC) and Bayesian Information Criterion (BIC) to select the best-fitting distribution, and performed sensitivity analysis to assess the robustness of the model results. Second, the study only focused on the two most serious AEs related to treatment, neglecting other potential AEs. As a result, there may be some degree of bias between the calculated costs and those in real-world settings. Third, several key parameters in the analysis, including utility scores, were obtained from the literature. However, a comprehensive search of existing studies did not identify utility scores specific to the Chinese population, which may impact the accuracy of the model results. Finally, this analysis was conducted while Rezvilutamide was still under patent protection. The pricing of generic equivalents may influence the cost-effectiveness analysis in future studies.

## Conclusion

In conclusion, our analysis indicates that rezvilutamide plus ADT is not cost-effective at the current price compared to bicalutamide plus ADT. However, it becomes cost-effective if the price of rezvilutamide is reduced by 14.29%.

## Data Availability

The original contributions presented in the study are included in the article/supplementary material, further inquiries can be directed to the corresponding authors.
